# Recommendations for Safe Dental Care: A Systematic Review of Clinical Practice Guidelines in the First Year of the COVID-19 Pandemic

**DOI:** 10.3390/ijerph181910059

**Published:** 2021-09-24

**Authors:** Naira Figueiredo Deana, Andrea Seiffert, Yanela Aravena-Rivas, Pablo Alonso-Coello, Patricia Muñoz-Millán, Gerardo Espinoza-Espinoza, Patricia Pineda, Carlos Zaror

**Affiliations:** 1 Department of Pediatric Dentistry and Orthodontics, Faculty of Dentistry, Universidad de La Frontera, Temuco 4781176, Chile; n.figueiredo01@ufromail.cl (N.F.D.); patricia.munoz@ufrontera.cl (P.M.-M.); patricia.pineda@ufrontera.cl (P.P.); 2 Center for Research in Epidemiology, Economics and Oral Public Health (CIEESPO), Faculty of Dentistry, Universidad de La Frontera, Temuco 4811230, Chile; yanela.aravena@ufrontera.cl (Y.A.-R.); gerardo.espinoza@ufrontera.cl (G.E.-E.); 3 Dental School, Faculty of Dentistry, Universidad de La Frontera, Temuco 4781176, Chile; andrea.seiffert@ufrontera.cl; 4 Iberoamerican Cochrane Centre, Biomedical Research Institute Sant Pau (IIB Sant Pau), 08025 Barcelona, Spain; PAlonso@santpau.cat; 5 CIBER Epidemiología y Salud Pública (CIBERESP), Barcelona, Spain; 6 Department of Public Health, Faculty of Medicine, Universidad de La Frontera, Temuco 4781135, Chile

**Keywords:** clinical practice guideline, dentistry, treatment decision-making, dental profession, COVID-19, infection control

## Abstract

In the context of a pandemic, the rapid development of clinical practice guidelines (CPGs) is critical to guide dental staff towards the safe provision of dental care; detailed knowledge of the recommendations will help to achieve the intended results. We carried out a systematic review of the recommendations in clinical practice guidelines (CPGs) on the provision of dental care issued during the first year of the COVID-19 pandemic. A systematic database search was performed in MEDLINE, EMBASE, LILACS, Epistemonikos, and Trip databases to identify documents with recommendations intended to minimize the risk of COVID-19 transmission during dental care. The selection process and data extraction were carried out by two researchers independently. The majority of CPGs recommended the use of rubber dam, high-volume evacuator, mouthwash prior to dental care, four-handed work, and mechanical barriers. The use of aerosol-generating equipment should be avoided whenever possible. In aerosol-generating procedures, the use of a N95 respirator (or similar) is recommended, in addition to a face protector, an impermeable disposable apron/gown, a cap, and gloves. The CPGs developed during the first year of the pandemic offer recommendations which guide dental staff in providing safe dental care, minimizing exposure to SARS-CoV-2 and reducing the risk of COVID-19 infection in the clinical environment. Such recommendations must, however, be updated as new evidence arises.

## 1. Introduction

Since late 2019, there has been a collective global effort to contain the COVID-19 pandemic. Various restrictive measures and social distancing have been put into effect [1], vaccines have been developed [2], and biosecurity measures in healthcare provision have been implemented [3,4,5]. Numerous clinical trials have been carried out to test the effectiveness of drugs to treat patients infected with SARS-CoV-2 [6]. So far, however, control of the virus has been difficult as it continues to infect thousands of people every day [7].

As a result of close contact with patients during dental care, dentists and dental assistants are exposed to a high risk of infection with COVID-19. Dental care providers are exposed to contaminated dental fluids, the propagation of bioaerosols, and contact with potentially contaminated instruments or surfaces [5,8]. Given the above risks, there was a substantial, general, and severe impact on dentistry in the early stages of the pandemic, when many countries restricted dental care to urgent and emergency cases only. Health authorities of the different countries took a similar stance at the beginning of the pandemic, recommending that dental care should be limited to emergency cases, and that elective dental procedures should be postponed [9]. Since pandemics tend to take longer than expected to decline [10], dentists have had to adapt and take the necessary measures to resume clinical care at the earliest opportunity [11], as elective treatments cannot be postponed indefinitely [10]. Currently, many countries have already allowed the opening of dental consults, restoring elective care. In this “new reality”, protection measures have been established to minimize the risk of COVID-19 transmission during dental clinical practice.

Although dentists are accustomed to the use of personal protection equipment and biosecurity measures [8], there was great uncertainty in the face of a new virus as to which steps would most effectively reduce the risk of infection during dental care. In an effort to provide guidance for dental workers, Health Ministries, government agencies, and the scientific community worked diligently to develop clinical practice guidelines (CPGs) in as short a time as possible to ensure safe dental care, with recommendations based on the best evidence available at the time. Previous reviews have summarized the recommendations of the clinical practice guidelines for dental care during the COVID-19 pandemic; however, they only included guidelines developed in the initial stages of the pandemic, before the reopening of dental services [8,12]. Therefore, our review complements these reviews by reporting on the recommendations provided at the time when normalizing dental care services was already in place.

In this study, we reviewed the dental CPGs developed during the first year of the COVID-19 pandemic to determine the principal recommendations for reducing exposure to SARS-CoV-2 and the risk of infection in the dental clinic setting.

## 2. Materials and Methods

### 2.1. Protocol and Registration

The systematic review was designed and reported following the guidelines of the Preferred Reporting Items of Systematic Reviews and Meta-Analyses (PRISMA) [13]. The protocol was published in Open Science Framework [14].

### 2.2. Eligibility Criteria

We included documents in English, Spanish, and Portuguese which were identified as guidelines providing recommendations for walk-in or emergency dental care in the context of the COVID-19 pandemic. A document was eligible if it was developed by national, regional, or local organizations; a team of experts; a panel of experts; or if it provided a list of authors involved in the process. We excluded clinical practice guidelines (CPGs) developed exclusively for patient special care, guidelines developed outside the context of the pandemic, previous versions of the same guideline, letters to the editor, and conference summaries.

### 2.3. Sources of Information

We performed a systematic search of the scientific literature to identify CPGs and protocols on procedures intended to minimize the risk of COVID-19 transmission during dental care. The search was conducted in MEDLINE, EMBASE, LILACS, CRD, Epistemonikos, and Trip databases up to December 2020. It was complemented by a manual search of guideline developer websites, CPG repositories, dental scientific societies, Health Ministries, and agencies related to COVID-19 management.

In addition, we examined the reference lists of the selected CPGs to identify other guidelines that met the inclusion criteria. We did not limit the search by date or publication status; however, only studies in English, Spanish, and Portuguese were included.

All search strategies and databases used are listed in supplementary material (Supplementary Material S1).

### 2.4. Selection of Guidelines

All references identified were extracted to an EndNote X9 database to facilitate their management and eliminate duplicate articles. Titles and abstracts of studies retrieved using the search strategy were screened independently by two authors to identify studies that potentially met the inclusion criteria. We obtained the full texts of all relevant and conceivably relevant studies meeting the inclusion criteria, as well as those for which there was insufficient data in the title and abstract to make a clear decision. Any disagreement between the two authors was resolved through discussion with a third reviewer. In the case of several documents from a single source, we included the most recent CPG.

### 2.5. Data Charting Process

One reviewer (C.Z., G.E.E., N.F.D., P.M.M., P.P., or Y.A.R.) extracted relevant data from eligible studies describing their main characteristics. An additional reviewer checked all the information extracted for accuracy (non-independent verification of data extraction). The following information was extracted from each article using a standardized, predefined data collection form: author, year, title, country, organization, language, setting, target population, and main recommendations.

### 2.6. Synthesis of the Results

The principal aspects of the guidelines and their main recommendations were reported descriptively and are presented in Figures and Tables. The recommendations were grouped by objective of the recommendation: triage and recommendations for patients; recommendations for the waiting room area; recommendations during dental procedures; recommendations after dental care; recommendations on dental treatment room ventilation recommendations for equipment and infrastructure; and personal protective measures for dentists, auxiliary staff, and patients.

## 3. Results

### 3.1. Literature Search

**Figure 1:**
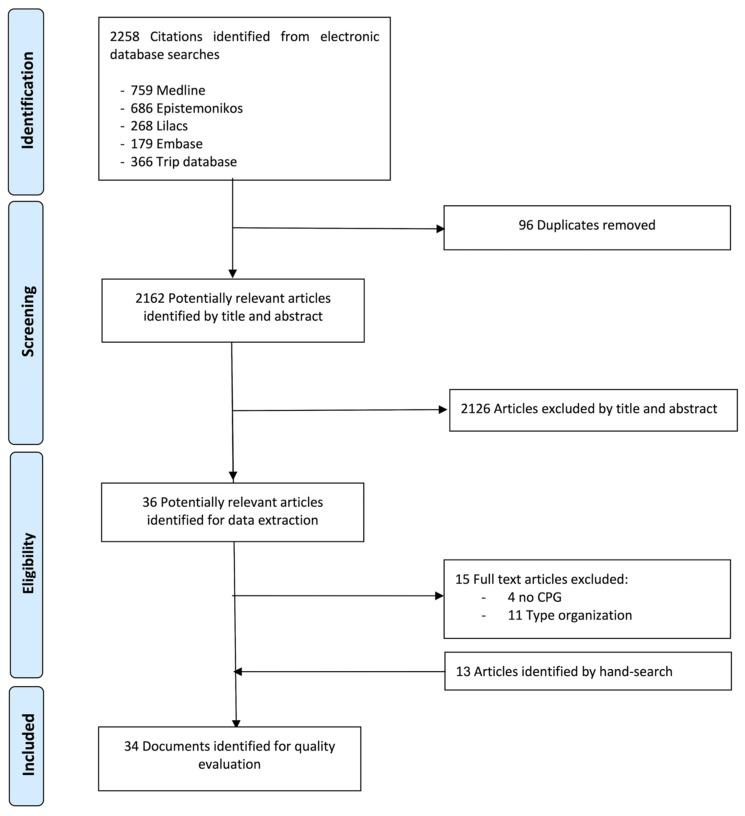
Flowchart of the CPGs included.

The search yielded 2258 references from the various databases, 96 of which were duplicates. A total of 2126 titles and abstracts were excluded as they did not meet the eligibility criteria. Furthermore, 36 documents were selected for full-text review, of which 15 were excluded after full-text review. In addition, thirteen articles were identified through other sources. Finally, 34 guidelines or protocols were included and their recommendations retrieved. Figure 1 shows the flow chart of the selection process.

### 3.2. Features of the Guidelines Included

Of the CPGs included, thirteen were in English [15,16,17,18,19,20,21,22,23,24,25,26,27], fourteen in Spanish [28,29,30,31,32,33,34,35,36,37,38,39,40,41], four in Portuguese [42,43,44,45], and three in other languages [46,47,48] (See Table 1). The developers were mainly Health Ministries or Government Departments and Agencies (19/34) and scientific societies (14/34); one guide was developed by a university (1/34). Following publication of the initial version of the guideline, a total of 17/34 guidelines were updated [15,16,17,18,20,22,23,24,26,31,32,39,42,43,44,45,46].

### 3.3. Summary of Recommendations

#### 3.3.1. Recommendations Regarding Triage and General Recommendations for Patients

Most of the CPGs recommended telephone triage (27/34) and in-person triage (26/34). Some guidelines indicated that, in addition to a preclinical survey when performing in-person triage, it is also necessary to measure temperature with an infra-red thermometer (13/34). A total of 70.6% of guidelines (24/34) made some type of recommendation regarding care of suspected or confirmed COVID-19 patients. For these patients, the most frequent recommendation was to perform emergency (16/34) care only; the least reported recommendation was not to perform any clinical attention (2/34). Most of the CPGs provided at least one recommendation related to general instructions for patients (31/34), frequently recommending hand-washing or cleaning with an alcohol-based disinfectant on arrival at the clinic, along with physical distancing (Table 2).

#### 3.3.2. Recommendations for the Waiting Room Area

Table 3 shows the most frequent recommendations for the waiting room. Most CPGs made at least one recommendation related to the waiting room (32/34), the most frequent being the implementation of physical distancing measures, followed by eliminating shared objects in the waiting room and the provision of alcohol hand-sanitizer. The least frequent recommendations made were related to placing a disinfectant doormat at the entrance to the clinic.

#### 3.3.3. Recommendations Regarding Personal Protective Equipment (PPE)

Only two CPGs did not make recommendations about the use of PPE during dental care. The use of respirators by dentists was recommended by 22/34 (64.7%) of CPGs in the case of treatments involving aerosol-generating procedures (AGP), 4/34 (11.8%) in cases involving AGP or confirmed COVID-19, and 6/34 (17.6%) guidelines recommended the use of respirators in all procedures. Some CPG developers recommended the use of additional protection to the respirator by use of either a surgical mask (4/34) or a face shield (1/34). The use of protective clothing, face protection (safety goggles/face shield), gloves and disposable cap was recommended for dentists. In total, 9/34 CPGs (26.5%) recommended that dentists should use all PPE at once (six elements) and 11/34 (32.3%) recommended the use of at least five PPE (no recommendation was made about the use of shoe covers) (Table 4).

In total, 8/34 (23.5%) CPGs recommended the use of a respirator by the dental assistant only in cases involving AGP. The use of protective clothing, face protection, a disposable cap, and a surgical mask was also recommended. A total of 14/34 (41.2%) CPGs made no recommendation as to the use of PPE by the dental assistant.

For patients entering the dental treatment room, the PPE recommendation was mainly related to the use of masks (24/34, 70.6%); however, the use of a disposable cap, shoe covers, outer clothing (gown or apron), and face protection (goggles) was also recommended. Only 7/34 (20.6%) of the guidelines made recommendations for the use of PPE for cleaning personnel, including the use of masks (8/34, 23.5%), rubber gloves (6/34, 17.6%), external protective clothing (4/34, 11.8%) such as boots and apron, and the use of some type of eye protection (5/34, 14.7%). For reception personnel, the most frequently recommended PPE was the surgical mask (14/34, 41.2%). It is worth noting that 18/34 (52.9%) of the guidelines did not make any recommendation with respect to the use of PPE by reception staff. All the guidelines recommended hand-washing or hand-cleaning with alcohol gel for professionals, staff, and patients.

#### 3.3.4. Recommendations for the Reduction in Aerosols during Dental Procedures

**Figure 2:**
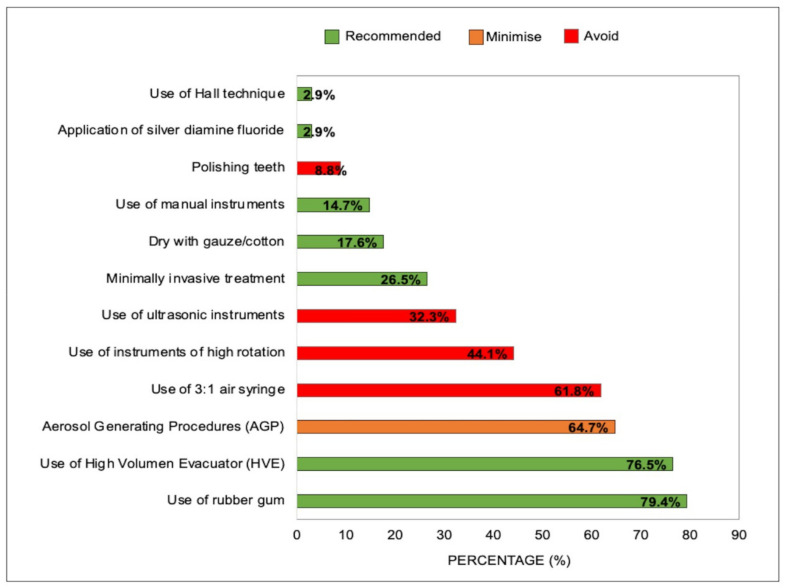
Recommendations for reducing aerosol production during dental procedures (*N* = 34 CPGs).

Procedures such as the use of high-volume evacuator (HVE) and the use of a rubber dam were widely recommended in order to reduce the generation of aerosols during dental care; the least frequent recommendations were to prioritize minimally invasive treatment alternatives, namely the application of diamine silver fluoride and the Hall Technique. Although most of the CPGs recommended avoiding the use of high rotation instruments, those that recommended their use suggested regulating the cooling output (Figure 2). Only 3/34 CPGs made no recommendations on how to reduce aerosol production during dental care.

#### 3.3.5. Recommendations to Minimize the Risk of Contamination

**Figure 3:**
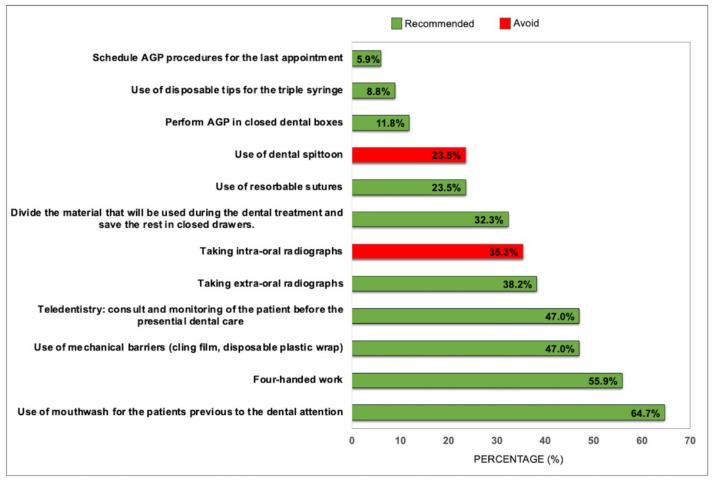
Recommendations to decrease contamination (*N* = 34 CPGs).

With regard to procedures aimed at reducing contamination, four-handed work and the use of mouthwashes prior to care were recommended by the vast majority of guidelines (Figure 3). Of the 22/34 CPGs that recommended the use of mouthwashes before dental care, the product most frequently recommended was hydrogen peroxide (18/22), followed by iodopovidone (9/22). A total of 8/34 CPGs (23.5%) did not report information regarding the use of mouthwashes and 4/34 CPGs (11.8%) did not recommend the use of oral rinses due to the lack of scientific evidence to support such a recommendation. The recommendations to use a surgical field, to use disposable tips for a triple syringe, and to avoid the use of a spittoon were the least frequently reported recommendations. Additional recommendations, such as performing treatment with the door closed (3/34, 8.8%), completing treatment during a single visit (4/34, 11.8%), performing AGP in isolated rooms (1/34, 2.9%), or programming AGP for the last appointment of the day (1/34, 2.9%,) are examples of further recommendations found in the guidelines. It is also worth noting that 2/34 (5.9%) of the guidelines did not report specific recommendations for this topic.

#### 3.3.6. Recommendations after Dental Care

Among the guidelines’ recommendations for actions following dental treatment were cleaning of material, equipment, and the clinical environment. CPGs also made recommendations on the type of cleaning material used, with 14/34 guidelines (41.2%) recommending the use of hypochlorite; 3 (8.8%) recommending the use of peracetic acid; and another 2 (5.9%) recommending the use of quaternary ammonium, glutaraldehyde, and phenolic compounds. For surface cleaning, 18 guidelines (52.9%) recommended the use of hypochlorite, 10 (29.4%) recommended alcohol, 7 (20.6%) recommended the use of soap or detergent, 3 (8.8%) recommended the use of quaternary ammonium, 1 (2.9%) recommended hydrogen peroxide, and 1 guideline (2.9%) recommended the use of phenolic compounds. Additionally, one guideline (2.9%) recommended the use of ozone or ultraviolet-C rays to disinfect surfaces.

#### 3.3.7. Recommendations on Dental Treatment Room Ventilation

In general, the guidelines recommended that dental clinics should maintain well-ventilated spaces (14/34, 41.2%), whenever possible with natural ventilation (4/34, 11.8%). The use of an air purifier with a HEPA filter (1/34, 2.9%), a portable air purifier with a HEPA filter (1/34, 2.9%), UV-C radiation, and aerosol disinfectant, were all recommended in dental treatment rooms without natural ventilation (1/34, 2.9%). The use of air conditioning was not recommended by 4/34 (11.8%) guidelines, and one guideline (3%) highlighted that air conditioning should be used only in the extraction mode. Negative pressure rooms with a HEPA filter were recommended by 4/34 (11.8%) guidelines for specific cases of AGP, suspected or confirmed COVID-19 patients, or in dental treatment rooms without a window.

After performing dental procedures, 6/34 guidelines (17.6%) recommended waiting 1 h before cleaning the dental treatment room to allow time for sedimentation of the aerosols. Two other guidelines (5.9%) recommended waiting 15 min, two guidelines (5.9%) recommended 3 h, and one (2.9%) recommended waiting 20 min. Other practices, such as requesting that the heating, ventilation and air conditioning (HVAC) to determine the number of air changes/hour (ACH), and performing the ACH calculation based on the Centers for Disease Control and Prevention, were also recommended by some guidelines. Two guidelines (5.9%) indicated that, following dental care, it was not necessary to wait any period of time before cleaning the dental treatment room.

#### 3.3.8. Recommendations for Bathrooms in Dental Clinics

Only 14/34 (41.2%) guidelines made recommendations with reference to dental clinic bathrooms. The main recommendations were to provide supplies for hand hygiene (14/34, 41.2%), to install hand washing and coughing guidance posters and signs, to maintain social distancing (8/34, 23.5%), and to ensure frequent disinfection of bathrooms (4/34, 11.8%).

#### 3.3.9. Recommendations for Treatment Rooms with More Than One Dental Chair

Only 5/34 (14.7%) guidelines made specific recommendations for dental care on open floors. The recommended distance between dental chairs varied between 1 meter (1/34, 2.9%), 2 meters (2/34, 5.9%), and 2.8 meters (2/34, 5.9%). One guideline (2.9%) made a recommendation on the height of the partition between chairs, of 2.6 meters. Finally, not performing AGP was recommended in 1/34 (2.9%) guideline.

## 4. Discussion

Clinical practice guidelines (CPGs) contain recommendations that inform users about the benefits and risks of any specific intervention or condition, to achieve the best health result [49]. Because of the various steps that have to be followed in the development of a CPG, the time required for preparation may vary between two and three years [50]. Consequently, CPG developers face a huge challenge when a new disease such as COVID-19 appears, given the need of health care providers for a rapid response, in short time-frames, based on high-quality evidence [14]. In the present study, we analyzed the dental CPGs developed in the first year of the pandemic to determine the principal recommendations made for safe dental practice. The majority of the CPGs analyzed in our study indicated the need for both telephone and in-person triage, made recommendations on the use of PPE, provided advice on procedures to reduce aerosols/bioaerosols contamination, and offered recommendations for the waiting room and the need for ventilation in the dental treatment room. However, not all CPGs addressed relevant aspects in the protection of dental personnel, such as adequate ventilation times after carrying out PGA, and the most appropriate type of ventilation for each type of dental consult setting. Furthermore, information on the need to avoid equipment, such as air-conditioning and air circulators, is scarce or missing altogether. Adequate ventilation is essential to reduce bioaerosols and, taking into account that most dental treatments require the use of aerosol-generating equipment, the recommendations for adequate ventilation acquire greater relevance and should be highlighted in future guidelines and updates.

Telephone and in-person triage were recommended by the majority of guidelines, since the interview helps to identify suspected/confirmed cases of COVID-19, preventing these patients from attending the clinic during the period when the disease is transmissible. However, one quarter of SARS-CoV-2 infections remain asymptomatic [51], rendering identification of these patients difficult in the initial triage; it is therefore essential to implement biosecurity measures during clinical practice. A frequently recommended biosecurity measure is to maintain physical distancing of 1 to 2 meters between patients, which is an effective way of reducing the risk of infection [52]. In a study on North American dentists, Araújo et al. [53] reported that telephone triage and encouraging physical distancing between patients were two measures widely adhered to by professionals, resulting in low COVID-19 transmission in dental environments.

Carrying out aerosol/bioaerosol-generating procedures exposes the dentist and dental assistants to a larger number of aerosol and bioaerosol particles, which can facilitate the risk of infection with airborne diseases, such as COVID-19 [54]. Equipment such as ultrasonic scalers, handpieces, and dental air polishers emit aerosols of different sizes [55], meaning that the concentrations of bioaerosols can vary according to the type of treatment: higher during grinding and caries treatment, and lower during sealing and ultrasonic scaling [54]. After dental procedures, spray contamination may extend to up to 36 inches, with high levels at up to 12 inches, and persist for 30 min after the AGP [56]. In the context of a pandemic, reducing the exposure of dental staff to these bioaerosols is essential. HVE and rubber dam are considered effective measures for reducing the level of contaminated particles in the air and their dissemination on surfaces in the dental treatment room [57]. Since this issue is of critical importance, most of the CPGs analyzed in our study recommended reducing AGP during dental treatment, combined with the use of methods for minimizing bioaerosols in the air, such as HVE. Most guidelines made recommendations for carrying out minimally invasive procedures and for the use of manual instruments instead of aerosol-generating equipment; there is a lack of consistent recommendations in the choice of procedures that do not generate aerosols during health emergencies.

The use of mouthwashes before dental care was also recommended by the majority of CPGs as a means of reducing the level of contamination by airborne particles emitted during dental treatment. Hydrogen peroxide and iodopovidone were recommended by most guidelines. Nevertheless, new evidence exists indicating that hydrogen peroxide does not reduce the viral load of COVID-19 [58,59]. The use of iodopovidone at concentrations of 1% and 7% can reduce the viral load of COVID-19 in human saliva and would therefore reduce the risk of transmission [58]. Ongoing updates of CPGs as new evidence emerges is essential to ensure that they are based on the best evidence available.

More than half the CPGs recommended keeping treatment rooms well ventilated, but few offered recommendations on the best type of ventilation. There was also no consensus on the interval that should be observed between patients, which varied from 15 min to 3 h. Adequate ventilation and air purification may be an important measure for reducing the level of bioaerosol contamination in the dental clinic [60,61]. Air purifiers with a HEPA filter [60], the HVAC system, and UV-C radiation [62] are complementary ventilation systems that facilitate reducing the risk of infection in health environments. In future CPG updates we would expect to find improved recommendations on ventilation systems, considering both their effectiveness in reducing contamination in the clinical environment and the costs associated with their implementation.

The use of PPE by health workers is one of the principal prevention measures against any airborne pathogen [63], and almost all CPGs made recommendations about PPE use. Although dentists are already accustomed to the use of standard PPE during clinical practice, they may be less accustomed to the use of more complete forms of PPE [64]; therefore, professionals should be trained to follow protocols for PPE use [65]. Most CPGs recommended the use of N95 respirators (or similar) for AGP or cases of patients with confirmed COVID-19; however, the evidence does not show conclusively that respirators are more effective in reducing COVID-19 during aerosol-generating medical procedures [66,67]. As a protection measure for dentists, CPGs recommended the use of respirators along with other PPE, such as face protection (face shield/goggles); however, the recommendations did not specify one type of eye protector over the other. The face shield may offer greater benefits as it protects a larger area of the face [68], but comparative studies are needed to determine which type of protector is more effective against COVID-19. The use of eye protection among healthcare workers is an effective measure for reducing the risk of infection with respiratory viruses [52], but as far as we know there are no specific studies involving dentists, and few studies carried out with SARS-CoV-2. In cases where there is little or no evidence on a new pathogen, or the evidence for a specific population is scarce, guideline developers can generate recommendations based on experts’ opinion [69] or indirect evidence [70,71]. However, when updating their CPGs, the developers should base their recommendations on direct evidence wherever possible, as this will increase the certainty of the evidence. Furthermore, it is important for recommendations in the guidelines to be supported by references, improving the rigor of development—which tends to be very poor in CPGs developed during the initial periods of pandemics [72].

The prevalence of COVID-19 among dentists is generally lower than in the general the population [53], ranging from 0.9% to 2.6% [53,73,74]. The low rates of COVID-19 among dentists could be related to their compliance with provisional biosecurity standards upon return to clinical practice [53]. Dentists have implemented a series of prevention measures, such as initial triage [75], routine use of PPE and the N95 respirator (or similar) [75], physical distancing among patients, disinfection of equipment between patients, and frequent surface cleaning [53]. Adherence to biosecurity measures may vary by sex and geographical region [73], and also depending on the availability of consumables [76]; all of these factors may increase or decrease the COVID-19 infection rate [73]. Having guidelines that use a transparent framework to qualify the body of evidence is critical, to allow users to understand the mechanism used in developing the recommendations.

### 4.1. Study Limitations

The main strength of this study is that information on the development of CPGs was obtained in a systematic search of the literature that included websites of developers and repositories of CPGs; however, there were certain limitations. Even though an extensive search was carried out, including grey literature, important guidelines in a language other than English, Spanish, or Portuguese may not have been included. Some guidelines were developed at the start of the pandemic and have not been updated. Considering the scarcity of evidence for this period, some recommendations may be superseded if current evidence is taken into account. Moreover, we included guidelines up to December 2020, and some of the recommendations may have been modified since then, as new evidence is constantly emerging. Finally, new CPGs with better quality evidence may have been developed more recently and, therefore, is not included in our selection. Nevertheless, we consider that the most important biosecurity measures for reducing the risk of infection with SARS-CoV-2 among dental staff have been reported, and are unlikely to suffer major changes in the short and medium term.

### 4.2. Clinical Implications and Future Prospects

The recommendations made in dental CPGs developed during the first year of the COVID-19 pandemic provide guidance for safe dental care. Adherence to these recommendations provides better protection against infection with COVID-19 among dental professionals and patients in the dental environment. The guidelines developed during this initial period offer biosecurity recommendations that can serve as a basis for future recommendations if new airborne diseases emerge. Many of the recommendations that have been adopted by dentists during this period of the pandemic may be implemented definitively, which would be a positive development considering the risk of outbreaks of new diseases. Because the great majority of dental treatments involve aerosol-generating equipment, dentists, dental services, and university dental care centers may face similar conditions to those encountered at the start of the COVID-19 pandemic whenever new airborne diseases emerge. It is therefore important to carry out new clinical studies in order to evaluate additional measures which may help reduce the exposure of dental staff to new diseases, thus reducing the risk of infection related with the clinical setting.

## 5. Conclusions

The main recommendations of the dental CPGs were oriented towards procedures to reduce the dissemination of bioaerosols, reduce the level of particle contamination in the air during dental care, and protect dental staff by the permanent use of PPE. The CPGs developed during the first year of the pandemic offer recommendations which guide dental staff in providing safe dental care, minimizing exposure to SARS-CoV-2, and reducing the risk of COVID-19 infection in the clinical environment; however, these need to be updated as new evidence emerges.

## Figures and Tables

**Table 1:** General information of the clinical practice guidelines.

Guide Title	Country	Organization	Language	Publication Date/Update	Reference
COVID-19. Recommendations for dental care. (COVID-19. Recomendaciones en Odontología)	Argentina	Health Ministry of Argentina	Spanish	10 June 2020 (1st Edition)	[28]
Manual of Good Practices in Biosecurity for dental environments. (Manual de buenas prácticas en bioseguridad para ambientes odontológicos)	Brazil	Federal Dental Council, ITI Brazil, ITI Mexico ILAPEO Faculty, Mexican Council of Oral and Maxillofacial Rehabilitation, AMP, APP, University of Concepción, Centre for Advanced Oral Rehabilitation and Implantology–Dental Faculty	Spanish, Portuguese	26 May 2020 (2nd Edition)	[46]
Biosecurity Guidelines. Technical adaptations in times of COVID-19. (Orientação de biossegurança. Adequações técnicas em tempos de COVID-19)	Brazil	São Paulo Regional Dental Council	Portuguese	July 2020 (2nd Edition)	[42]
Technical note GVIMS/GGTES/ANVISA n. 04/2020 Guidelines for health services: prevention and control measures that should be adopted during attention to suspected or confirmed cases of infection by the new coronavírus (SARS-CoV-2). (Nota técnica GVIMS/GGTES/ANVISA n. 04/2020 Orientações para serviços de saúde: medidas de prevenção e controle que devem ser adotadas durante a assistência aos casos suspeitos ou confirmados de infeccção pelo novo coronavírus (SARS-CoV-2)).	Brazil	National Health Vigilance Agency-ANVISA	Portuguese	27 December 2020 (5th Edition)	[43]
Joint technical note nº014/2020-DAPS/DIVS/DIVE/SES/COMSES/SC. Recommendations for dental attention to SUS in Santa Catarina (Nota técnica conjunta nº014/2020-DAPS/DIVS/DIVE/SES/COMSES/SC. Recomendações ao atendimento odontológico do SUS em Santa Catarina)	Brazil	Health Secretary of Santa Catarina	Portuguese	15 October 2020 (2st Edition)	[44]
CDSS Alert–COVID-19 Pandemic: IPC Interim Protocol Update.	Canada	The College of Dental Surgeons of Saskatchewan	English	7 December 2020 (2nd Edition)	[18]
Return-to-practice office manual. Adapting the dental office to the COVID-19 Pandemic	Canada	Dental Association of Prince Edward Island. Member of the Canadian Dental Association [19]	English	10 May 2020 (1st Edition)	[19]
Rules for attention during the COVID-19 epidemic. (Normativa de atención contingencia COVID-19)	Chile	Chilean Dentistry Teaching Association [29]	Spanish	1 June 2020 (1st Edition)	[29]
Guidelines for dental attention in COVID-19 Phase IV. (Orientaciones para atención odontológica en fase IV COVID-19)	Chile	Public Health Undersecretary, Disease Prevention and Control Division, Oral Health Department, Health Ministry, Government of Chile	Spanish	17 May 2020	[30]
LS-SS-008. Technical Guidelines for the prevention and containment of COVID-19 for dentists and auxiliary staff in Costa Rica. (LS-SS-008. Lineamiento técnico para la prevención y contención de COVID-19 para odontólogos y personal auxiliar de Costa Rica)	Costa Rica	Health Ministry, Costa Rican Social Security Fund, College of Dental surgeons, Justice and Peace Ministry	Spanish	12 August 2020 (2nd Edition)	[31]
Protocol for dental attention in urgent and emergency cases during the COVID–19 health emergency.(Protocolo para atención odontológica en emergencias y urgencias odontológicas durante la emergencia sanitaria por COVID–19)	Ecuador	Health Ministry, Ecuadorian Institute of Social Security, Armed Forces, Ecuadorian Society of Public Health, Ecuadorian Dental Federation	Spanish	May 2020 (3rd Edition)	[32]
Technical guidelines for dental attention after the COVID-19 emergency. (Lineamientos técnicos para la atención odontológica posterior a la emergencia por COVID-19)	El Salvador	Health Ministry, Government of El Salvador	Spanish	14 June 2020 (1st Edition)	[33]
Standard operating procedure. Transition to recovery. A phased transition for dental practices towards the resumption of the full range of dental provision	England	Office of Chief Dental Officer England (OCDO), National Health Services (NHS)	English	28 August 2020 (3rd Edition)	[15]
COVID-19 guidance and standard operating procedure. For the provision of urgent dental care in primary care dental settings and designated urgent dental care provider sites	England	National Health Services (NHS)	English	28 August 2020 (3rd Edition)	[16]
Dental Biosecurity Protocol with emphasis on COVID-19. (Protocolo de Bioseguridad Odontológica con énfasis en COVID-19)	Guatemala	College of Stomatology of Guatemala	Spanish	May 2020 (1st Edition)	[41]
Advisory: Dental Clinics Protocols	India	Dental Council of India	English	7 May 2020 (1st Edition)	[25]
Guidelines for oral health services at COVID-19 Alert Level 1	New Zealand	Ministry of Health, Dental Council	English	December 2020 (6th Edition)	[26]
Guidelines for dental attention to patients with suspected or confirmed COVID-19 in health facilities. (Guía para el manejo odontológico de pacientes sospechosos o confirmados por COVID-19 en las instalaciones de salud)	Panama	Health Ministry of Panama	Spanish	March 2020 (1st Edition)	[34]
Protocol for dental attention during gradual return. (Protocolo para atención Odontológica de retorno gradual)	Paraguay	Ministry of Public Health and Social Welfare, National Government	Spanish	7 May 2020 (1st Edition)	[35]
Biosecurity protocol for dental surgeons during and after the COVID-19 pandemic. (Protocolo de bioseguridad para el cirujano dentista durante y post pandemia COVID-19)	Peru	Dental College of Peru, National Administrative Council	Spanish	26 April 2020 (1st Edition)	[36]
Clinical recommendations for the execution of dental-stomatological procedures in the context of the COVID-19 pandemic. (Recomendaciones clínicas para realizar procedimientos en odontoestomatología en el contexto de pandemia por COVID-19)	Peru	Institute for Technology Evaluation in Health and Research (IETSI)	Spanish	13 April 2020 (1st Edition)	[37]
COVID-19: Procedures in primary oral health care services, clinics or consultancies, public and private sectors. (COVID-19: Procedimentos em clínicas, consultórios ou serviços de saúde oral dos cuidados de saúde primários, setor social e privado)	Portugal	Order of Dental Surgeons, National Oral Health Promotion Programme, National Programme for the Prevention and Control of Infections and Microbial Resistance	Portuguese	27 July 2020 (2nd Edition)	[45]
Guidelines for dental care provision during the COVID-19 pandemic	Saudi Arabia	University	English	7 April 2020 (1st Edition)	[27]
Novel coronavirus (COVID-19) Guidance for primary care	Scotland	Public Health Scotland, National Health Services Scotland (NHS)	English	17 September 2020 (12th Edition)	[20]
Resuming General Dental Services Following COVID-19 Shutdown.A guide and implementation tools for general dental practice. For Phases 2 and 3 of dental services remobilisation	Scotland	Scottish Dental Clinical Effectiveness Programme (SDCEP)	English	12 June 2020 (1st Edition)	[21]
Strategic Action Plan for the COVID-19 scale-back period. (Plan Estratégico de acción para el periodo de desescalada COVID-19)	Spain	Dental Council of Spain	Spanish	2 May 2020 (2nd Edition)	[39]
Recommendations by the Spanish Society of Epidemiology and Oral Public Health (SESPO) for the healthcare adaptation of public health dental clinics in Spain during the COVID-19 pandemic	Spain	Spanish Society of Epidemiology and Oral Public Health (SESPO)	Spanish	December 2020 (1st Edition)	[38]
COVID-19 infection prevention and control measures for primary care, including general practitioner practices, dental clinics and pharmacy settings: first update	Europe	European Centre for Disease Prevention and Control (ECDC)	English	19 October 2020 (2nd Edition)	[24]
Implications of COVID-19 for the safe management of general dental practice. A practical guide	UK	College of General Dentistry, Faculty of General Dental Practice	English	2 October 2020 (2nd Edition)	[17]
Health Ministry recommendations for dental professionals and dental hygienists. (Recomendaciones del ministerio de salud pública para profesionales odontólogos e higienistas dentales)	Uruguay	Public Health Ministry of Uruguay	Spanish	27 March 2020 (1st Edition)	[40]
Guidance for Dental Settings. Interim Infection Prevention and Control Guidance for Dental Settings During the coronavirus disease 2019 (COVID-19) Pandemic	USA	Center for Disease Control and Prevention	English	4 December 2020 (2nd Edition)	[22]
Return to Work Interim Guidance Toolkit	USA	American Dental Association (ADA)	English	23 July 2020 (2nd Edition)	[23]
Attention path for Odontopediatric procedures during shutdown or quarantine stages of the COVID-19 pandemic. (Ruta de atención para procedimientos de Odontología Pediátrica durante la etapa de confinamiento o cuarentena de la pandemia COVID-19)	-	Latin American Association of Odontopediatrics (ALOP)	English, Spanish, Portuguese	11 April 2020 (1st Edition)	[47]
Considerations for the provision of essential oral health services in the context of COVID-19: interim guidance, 3 August 2020	-	World Health Organization (WHO)	Arabic, Chinese, English, French, Portuguese, Russian, Spanish	3 August 2020 (1st Edition)	[48]

**Table 2:** Telephone triage, in-person triage, and general recommendations for the patients.

Topic	Recommendation	*N* (%)
1. Telephone triage: before dental care	Assess and record with a survey the symptoms of suspected and confirmed cases of COVID-19, before they attend for dental care.	27 (79.4%)
2. In-person triage: once the patient arrives at the clinic, before dental care	Assess and record with a survey the symptoms of suspected and confirmed cases of COVID-19.	26 (76.5%)
	once the patient arrives for dental care. Assess and record with a survey the symptoms of suspected and confirmed cases of COVID-19, associated with taking the patient’s temperature.	13 (38.2%)
3. Patients with confirmed or suspected COVID-19	Only urgent or emergency treatment	16 (47.0%)
	Delay the dental treatment	13 (38.2%)
	Refer the patient to a health centre that receives and treats patients with confirmed or suspected COVID-19.	9 (26.5%)
	Treat the patient in an isolated room.	8 (23.5%)
	Treat the patient at the end of the day.	4 (11.8%)
	Treat these patients on a different day	4 (11.8%)
	Do not provide clinical services	2 (5.9%)
4. Presence of accompanying persons	Patients not requiring assistance should attend the clinic alone. Only patients who require assistance, such as minors, patients with special needs and elderly patients, should be accompanied.	24 (70.6%)
5. General guidelines for patients	Wash hands or use alcohol-based disinfectant.	28 (82.3%)
	Physical distancing (between patients)	19 (55.9%)
	Remove personal ornaments	11 (32.3%)
	Avoid bringing personal objects and avoid the use of cellphones	7 (20.6%)
	Hair should be tied back	4 (11.8%)
	Do not brush teeth in the dental clinic	4 (11.8%)
	Brush teeth in the dental clinic before dental care	4 (11.8%)
6. Returning home	Give guidance for safe return home	1 (2.9%)

**Table 3:** Recommendations for the waiting room.

Recommendation	*N* (%)
Physical separation measures to maintain social distancing: separate chairs (1 to 2 m between chairs), remove unnecessary chairs to encourage social distancing between patients, decrease the maximum capacity of the waiting room, delineate physical spaces with tables and chairs to define the flow zones.	29 (85.3%)
Take away all shared objects from the waiting room: magazines, books, informative booklets, table games or toys.	26 (76.5%)
Provide alcohol-based hand disinfectant at the entrance to the waiting room.	23 (67.6%)
Install visual alerts in waiting room areas making recommendations about hand-washing protocols, social distancing and coughing.	17 (50.0%)
Install a transparent panel in the reception area to separate staff from patients.	17 (50.0%)
Install signs indicating the safe social distance in the reception area.	12 (35.3%)
Install a disinfectant doormat at the entrance to the clinic.	7 (20.6%)

**Table 4:** Recommendations for use of personal protective equipment (PPE) by dentists, dental assistants, patients, reception staff, and cleaning staff.

Population	Respirator N95 or Similar	Surgical Mask	Gloves	Disposable Cap	Shoe Covers	External Protective Clothing (Disposable Apron/Gown)	Face Protection (Goggles/Face Shield)	CPGs without Report
	*N* (%)	*N* (%)	*N* (%)	*N* (%)	*N* (%)	*N* (%)	*N* (%)	*N* (%)
Dentist	32 (94.1%)	24 (70.6%)	31 (91.2%)	23 (67.6%)	11 (32.3%)	32 (94.1%)	32 (94.1%)	2 (5.9%)
Dental assistant	13 (38.2%)	10 (29.4%)	12 (35.3%)	13 (38.2%)	3 (8.8%)	16 (47.0%)	16 (47.0%)	15 (44.1%)
Patients	-	24 (70.6%)	-	7 (20.6%)	6 (17.6%)	11 (32.3%)	11 (32.3%)	8 (23.5%)
Reception staff	-	14 (41.2%)	-	7 (20.6%)	1 (2.9%)	4 (11.8%)	9 (26.5%)	18 (52.9%)
Cleaning staff	-	8 (23.5%)	6 (17.6%)	3 (8.8%)	2 (5.9%)	5 (14.7%)	7 (20.6%)	27 (79.4%)

## Data Availability

The data and materials supporting the conclusions of this manuscript are included in the article.

## Supplementary Materials

The following are available online at https://www.mdpi.com/article/10.3390/ijerph181910059/s1. Supplementary Material S1. Search strategy used in each database.
