# Supplementary material for: Recommendations for Safe Dental Care: A Systematic Review of Clinical Practice Guidelines in the First Year of the COVID-19 Pandemic
Source: Int J Environ Res Public Health. 2021 Sep 24;18(19):10059. doi: 10.3390/ijerph181910059 (PMC8508467; doi:10.3390/ijerph181910059)
Supplement: Supplementary file 1 [file ijerph-18-10059-s001.zip › ijerph-1353238-supplementary.pdf]

SEARCH STRATEGY USED IN EACH DATABASE

MEDLINE

Search ((((((((((((((((((covid-19) OR covid19) OR sars-cov 2) OR covid*) OR cov2) OR coronaviridae) OR MERS-cov) OR sars cov 2) OR sars2) OR coronavirus) OR "Coronavirus"[Mesh]) OR "Coronavirus Infections"[Mesh]) OR sars-cov-2) OR "severe acute respiratory syndrome coronavirus 2" [Supplementary Concept]) OR mers cov) OR "Middle East Respiratory Syndrome Coronavirus"[Mesh])) AND ((((((((oral) OR dental)) AND ((((((prevention) OR care) OR health) OR procedure*) OR treatment*) OR "Health"[Mesh]))) OR dentistry) OR "Dentistry"[Mesh]) OR "Oral Health"[Mesh]) OR "Dental Care"[Mesh])) AND ((((((((((guideline*) OR recommendation*) OR protocol*) OR consensus) OR practice guideline) OR guidance) OR "Guideline" [Publication Type]) OR "Practice Guideline" [Publication Type]) OR "Health Planning Guidelines"[Mesh]) OR "Consensus"[Mesh]) OR "Guidelines as Topic"[Mesh])))

EMBASE

('coronavirinae'/exp OR 'coronavirus infection'/exp OR 'sars coronavirus'/exp OR 'coronavirus'/exp OR coronavirus OR 'middle east respiratory syndrome coronavirus'/exp OR 'covid 19' OR 'sars cov 2' OR covid19 OR 'mers cov' OR covid* OR cov2 OR 'cov 2') AND (dentistry OR 'dentistry'/exp OR 'mouth hygiene'/exp OR 'dental procedure'/exp OR 'preventive dentistry'/exp OR ((prevention OR care OR health OR 'health'/exp OR procedure* OR treatment*) AND (oral OR dental))) ND (guideline* OR 'guideline'/exp OR 'practice guideline'/exp OR 'consensus development'/exp OR 'consensus'/exp OR recommendation* OR consensus OR protocol* OR 'protocol'/exp)

LILACS

(MH:Dentistry OR TW:dentistry OR MH:"Oral Health" OR MH:"Dental Care" OR TW:dental OR TW:tooth OR TW: dental care OR TW:dental procedure$ OR TW:dental treatment OR TW:oral health) AND (MH:Coronavirus OR TW:coronavirus OR MH:"Coronaviridae Infections" OR TW:covid19 OR TW:covid-19 OR TW:sars-cov-2 OR TW: sars-covid-2 OR TW:cov2 OR TW:mers-cov-2) AND (TW:protocol$ OR TW:recommendation$ OR TW:Consensus OR TW:guideline$ OR TW:guidance OR MH:Guideline OR MH:"Practice Guideline")

EPISTEMONIKOS

(title:(coronavirus OR coronavirinae OR covid-19 OR covid19 OR sars-cov-2 OR Coronaviridae Infections OR mers-cov-2) OR abstract:(coronavirus OR coronavirinae OR covid-19 OR covid19 OR sar-cov-2 OR Coronaviridae Infections OR mers-cov-2)) AND (title:(dentistry OR oral health OR dental OR dental care OR dental procedure* OR dental treatment*) OR abstract:(dentistry OR oral health OR dental care OR dental OR dental care OR dental procedure* OR dental treatment*)) [Filters: classification=broad-synthesis]

Límite: Broad synthesis / Guideline

TRIP DATABASE

((covid-19 OR coronavirus OR coronavirinae OR Coronaviridae Infections OR sars-cov-2 OR mers cov OR cov2) AND (dentistry OR oral health OR dental OR dental care OR dental procedure OR dental treatment OR dental health OR oral care)) from:2020 to:2020 Límite: Guideline

WEBSITES

Guidelines developers

- National Institute for Health and Care Excellence (http://www.nice.org.uk)
- Scottish Intercollegiate Guidelines Network (http://www.sign.ac.uk)
- Guía Salud (https://portal.guiasalud.es)
- CMA Infobase: Clinical Practice Guidelines Database (CPGs) (https://joulecma.ca/cpg/homepage)
- Australian Clinical Practice Guideline (https://www.clinicalguidelines.gov.au)
- New Zealand Guidelines (https://www.health.govt.nz/about-ministry/ministry-health-websites/new-zealand-guidelines-group)
- Scottish Dental Clinical Effectiveness Programme (http://www.sdcep.org.uk)
- EBM Guidelines (https://www.ebm-guidelines.com/dtk/ebmg/home)
- IECS: Instituto de Efectividad Clínica y Sanitaria (https://www.iecs.org.ar).

CPGs compiler entities

- National Guideline Clearinghouse (http://www.guideline.gov)
- Guideline International Networks (GIN): (http://www.g-i-n.net/library/international-guidelines-library)
- ECRI Guidelines Trust (https://guidelines.ecri.org/)
- NeLH Guidelines Finder (http://libraries.nelh.nhs.uk/guidelinesFinder/)
- Agency for Healthcare Research and Quality (AHRQ) https://www.ahrq.gov/research/findings/evidence-based-reports/search.html)
- Guideline Central(https://www.guidelinecentral.com/)
- The Alliance for the Implementation of Clinical Practice Guidelines (https://aicpg.org/)
- Scottish dental (https://www.scottishdental.org/professionals/guidelines/)

Scientific Societies and Health Organizations

- World health Organization (OMS) (https://www.who.int/es)
- Organización Panamericana de la Salud (OPS) (https://www.paho.org/en)

- American Dental Association (ADA) (https://www.ada.org/en)
- FDI World Dental Association (https://www.fdiworlddental.org)
- International Association for Dental Research (IADR)(https://www.iadr.org)
- American Academy of Pediatric Dentistry (AAPD) (http://www.aapd.org/)
- Australian and New Zealand Society of Pediatric Dentistry (ANZSPD) (https://www.anzspd.org.au/)
- European Academy of Paediatric Dentistry (EAPD) (http://www.eapd.gr/)
- International Association of Paediatric Dentistry (IAPD) (http://www.iapdworld.org/)
- Pediatric Dentistry Association of Asia (PDAA) (http://pdaasia.org/)

Ministries of Health

- Africa: Nigeria (http://www.fmh.gov.ng), South Africa (http://www.doh.gov.za/), Sudan (http://www.fmoh.gov.sd/), Tanzania (http://www.moh.go.tz/) and Uganda (http://health.go.ug/mohweb/).
- America: Argentina (https://www.argentina.gob.ar/salud), Brasil (https://saude.gov.br), Bolivia (https://www.minsalud.gob.bo), Chile (http://www.minsal.cl), Colombia (https://www.minsalud.gov.co/portada-covid- 19.html), Costa Rica (http://www.ministeriodesalud.go.cr/), Cuba (http://www.sld.cu/), Dominican Republic (http://www.salud.gob.do/), El Salvador (http://www.salud.gob.sv/), Nicaragua and (http://www.minsa.gob.ni/), Perú (https://www.gob.pe/minsa/), Trinidad and Tobago (http://www.health.gov.tt/) and United States of America (http://www.hhs.gov/).
- Asia: Bhutan (http://www.health.gov.bt/), Cambodia (http://www.moh.gov.kh/), China (http://www.moh.gov.cn), Indonesia (http://www.depkes.go.id), Iraq
(http://www.moh.gov.iq),
(http://www.mhlw.go.jp/),
(http://www.mohp.gov.np),
(www.pakistan.gov.pk), Singapore
(http://www.health.gov.lk/), Thailand (http://eng.moph.go.th/) and Turkey (http://www.sb.gov.tr/).

- Europe: Austria (http://www.bmg.gv.at/), Belgium (http://www.health.belgium.be), Cyprus (http://www.moh.gov.cy), Denmark (http://www.sst.dk/), England (http://www.dh.gov.uk), France (http://www.sante.gouv.fr/), Germany (http://www.bmg.bund.de), Greece (http://www.yyka.gov.gr/), Iceland (http://www.velferdarraduneyti.is/), Ireland (http://www.dohc.ie), Italy (http://www.salute.gov.it/), Netherlands (http://www.government.nl/ministries/vws), Norway (http://www.regjeringen.no), Portugal (http://www.portaldasaude.pt), Spain (http://www.msc.es/), Sweden (http://www.folktandvardenstockholm.se/) and Switzerland (http://www.bag.admin.ch).
- Oceania: Australia (http://www.health.gov.au/) and New Zealand (http://www.health.govt.nz).

Websites of institutions related to the management of COVID-19

- China, USA and European Centers for Disease Control and Prevention (CDC)
- Members of the International Society of antimicrobial chemotherapy (89 members)
- Infectious Disease Society of America (IDSA)
- Israel Malaysia (http://www.old.health.gov.il), (http://ohd.moh.gov.my/v2/),
- Oman (http://www.moh.gov.om/), (http://www.ndc.com.sg), Sri Nepal Pakistan Lanka
- Sociedad Española de Enfermedades Infecciosas y Microbiologia Clínica (SEIMC)
- The International Society of Infectious disease (ISID)
- The European Society of Clinical Microbiology and infectious diseases (ESCMID)
- National Health Portal India (NHP)
- Agencia Nacional de Vigilancia Sanitaria (ANVISA)
- Public Health Agency of Canada (PHAC)
- Public Health England (PHE)
- Australian Government Department of Health (DOH)
- New South Wales Department of Health (NSW Health)
- National Institute of Infectious Diseases (NIID)- Japan
